# Supplementary material for: Retinoic Acid Grafted to Hyaluronic Acid Activates Retinoid Gene Expression and Removes Cholesterol from Cellular Membranes
Source: Biomolecules. 2022 Jan 25;12(2):200. doi: 10.3390/biom12020200 (PMC8961547; doi:10.3390/biom12020200)
Supplement: Supplementary file 1 [file biomolecules-12-00200-s001.zip › biomolecules-1535448-supplementary.pdf]

Spectroscopic characterization of HA-atRA

The chemical structure of the prepared conjugate HA-atRA was confirmed by a combination of spectroscopic techniques (1H-NMR, HSQC and DOSY). The 1H NMR spectra measured in D2O, had shown the typical proton chemical shifts of HA (Figure XX). The signal at 2.0 ppm belongs to -NHCOCH3, skeletal signals of at 3.4–3.9, and anomeric resonances from 4.47 to 4.37 ppm. Remaining signals detected in the spectrum can be attributed to CH3, CH2; and vinyl functional groups of retinoyl moiety, at 0.97; 1.41, 1.55, 1,68, 2.25; 5.58,6.28,6.38 and 7.31 ppm, respectively. The signals located at 7.48, 7.62 and 8.0 correspond to the benzoylation of HA produced due to mixed anhydride intermediate. The formation of a covalent bond between atRA and HA was established by diffusion ordered NMR spectroscopy (data not shown). Furthermore, HA-atRA exhibited a strong UV absorption band at ʎmax= 341 nm (Figure x2). UV-vis is useful for the characterization of low substituted HA-atRA. The method is based on a calibration curve and uses retinoic acid as a standard. The purity of the conjugate was confirmed by HPLC. Thus, retinoic acid in the samples was determined to be less than 0.035 % wt.

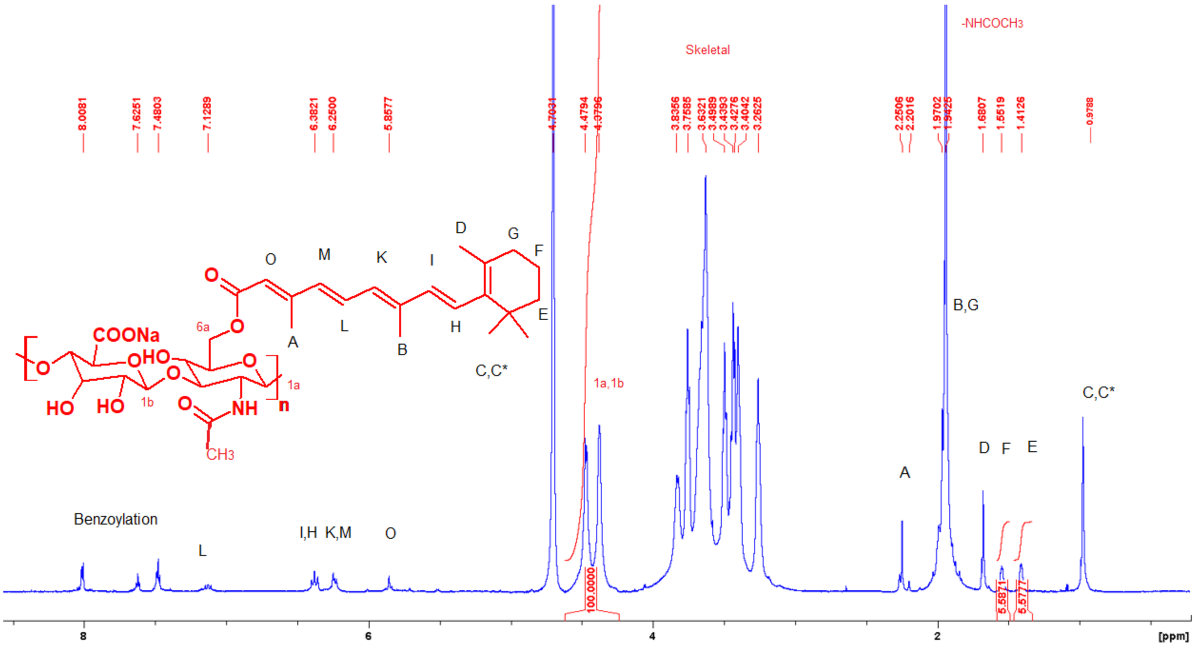

Figure S1. ^{1}HNMR of HA-atRA measured in D_2O (DS=5.5% Mw=15,000 g/mol).

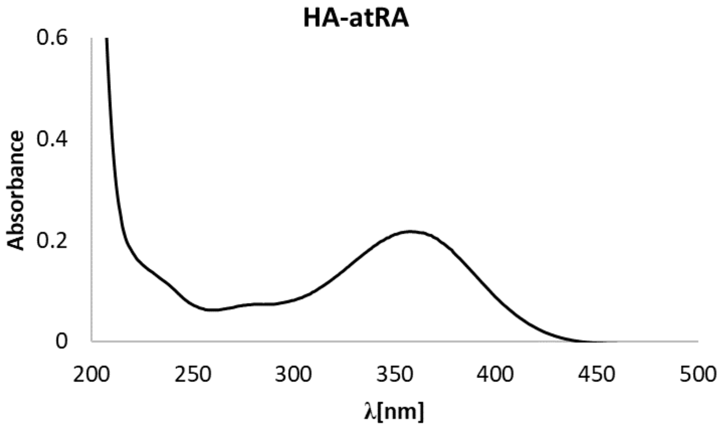

Figure S2. UV-Vis spectra of HA-atRA (DS = 2.21 %, Mw= 15,000 g/mol).

Table S1. Genes upregulated in NHDF fibroblasts treated with HA-atRA or atRA by more than 200 % relative to control (log2 fold change (FC) > 1) and $p < 0.05$ (Benjamini-Hochberg correction for multiple testing) in one or both treatment groups. Log2 FC 1 corresponds to 200 % upregulation, log2 FC 2 = 400 %, log2 FC 3 = 800 % etc.

Gene	Log2 FC		Difference of HA-atRA and atRA log2 FC	In GO:0006695 Cholesterol biosynthetic process
	HA-atRA	atRA		
DHRS3	5.0	4.3	0.6	
HMGCS1	3.5	0.0	3.5	Yes
LXN	3.4	3.8	-0.4	
RARRES1	3.0	3.3	-0.3	
CFI	2.7	3.1	-0.3	
RGS2	2.5	2.0	0.5	
SULF1	2.5	2.4	0.0	
FDFT1	2.1	0.0	2.0	Yes
IDI1	2.1	-0.2	2.2	Yes
SQLE	2.0	0.0	2.1	Yes
DHCR7	2.0	0.4	1.6	Yes
HOXA5	1.9	1.2	0.7	
KRT18P55	1.9	2.2	-0.3	
DOCK11	1.9	1.8	0.1	
MSMO1	1.9	-0.1	2.0	Yes
ARHGAP20	1.8	1.9	0.0	
HMGCR	1.8	-0.5	2.3	Yes
KRT18	1.8	2.2	-0.4	
HOXA3	1.8	1.4	0.4	
MVK	1.8	0.0	1.7	Yes
MEST	1.8	0.9	0.9	
ACAT2	1.7	-0.1	1.9	Yes
FASN	1.6	0.0	1.6	
FDPSL2A	1.5	0.3	1.2	
SMAD3	1.4	1.5	-0.1	
GPRC5A	1.4	1.2	0.3	
CTSH	1.4	1.4	0.0	
LIPA	1.4	0.5	0.9	
IDH1	1.3	0.5	0.9	
DHCR24	1.3	-0.4	1.7	Yes
PLAT	1.3	1.5	-0.2	
TMEM97	1.3	-0.3	1.6	
GALNT12	1.2	1.6	-0.4	
WNT2B	1.2	1.6	-0.3	
EPHA4	1.2	0.8	0.4	
HSD17B7	1.2	-0.1	1.3	Yes
PTPRJ	1.2	1.1	0.0	
RHOBTB3	1.1	0.9	0.2	

EBP	1.1	-0.2	1.3	Yes
DRAM1	1.1	1.4	-0.3	
IFIT3	1.0	1.2	-0.2	
FAM65B	1.0	0.8	0.2	
TANC1	1.0	0.9	0.1	
COLEC12	2.8	2.9	-0.1	
PDE5A	2.5	2.7	-0.2	
CCL2	2.3	2.4	0.0	
EPAS1	1.5	2.3	-0.7	
IL1B	1.8	2.2	-0.4	
PTGIS	1.6	2.1	-0.5	
STRA6	1.3	2.0	-0.7	
RCAN2	2.4	1.8	0.5	
ANGPTL4	-1.1	1.7	-2.8	
FBXO32	1.0	1.7	-0.7	
GPRC5B	1.0	1.7	-0.7	
GATA6	1.7	1.7	0.0	
DDIT4	1.6	1.6	0.0	
FAP	1.2	1.5	-0.3	
C1R	1.1	1.5	-0.5	
RBP1	1.7	1.5	0.2	
PSMB9	0.9	1.5	-0.6	
SYNPO2	1.0	1.5	-0.5	
PIM1	0.5	1.5	-1.0	
TMTC2	1.4	1.5	-0.1	
METTL7A	1.6	1.5	0.1	
RDH10	1.4	1.4	0.0	
IGFBP6	1.1	1.4	-0.3	
C1S	0.9	1.4	-0.5	
TRIL	1.1	1.4	-0.3	
STAR	2.1	1.3	0.7	
CYP1B1	1.2	1.3	-0.1	
PTGES	1.0	1.3	-0.3	
TRIM22	0.9	1.3	-0.4	
ABCA1	-0.1	1.3	-1.4	
PPP1R12B	0.3	1.3	-1.0	
IRF1	1.1	1.3	-0.2	
PAMR1	1.4	1.2	0.2	
QPRT	1.8	1.2	0.6	
MGP	1.0	1.2	-0.2	
NMT2	0.9	1.2	-0.3	
VCAN	0.8	1.2	-0.3	
ARHGEF15	0.3	1.2	-0.9	
PBX1	0.9	1.2	-0.2	
RARB	1.3	1.2	0.2	
CCL26	1.2	1.2	0.1	

WARS	1.0	1.1	-0.1
PAPPA	0.9	1.1	-0.3
IGFBP3	0.6	1.1	-0.5
CRABP2	0.8	1.1	-0.3
LUM	1.1	1.1	0.0
LTBP3	0.8	1.1	-0.3
ASNS	0.7	1.1	-0.4
FAM43A	1.2	1.1	0.1
TGM2	0.8	1.1	-0.3
DAAM1	1.0	1.1	-0.1
PKIA	0.6	1.1	-0.5
BCL3	0.7	1.1	-0.3
SLC12A8	0.7	1.1	-0.3
SLC15A3	0.7	1.1	-0.4
LOXL2	0.6	1.0	-0.5
SULF2	0.8	1.0	-0.2
GJA1	0.7	1.0	-0.4
HIC1	0.6	1.0	-0.4
DACT1	1.1	1.0	0.0
KLHL24	1.4	1.0	0.3
UBE2L6	0.7	1.0	-0.4
CFH	0.6	1.0	-0.4
KRT8P12	0.6	1.0	-0.4
PLK2	0.9	1.0	-0.2
LHFPL2	0.9	1.0	-0.1

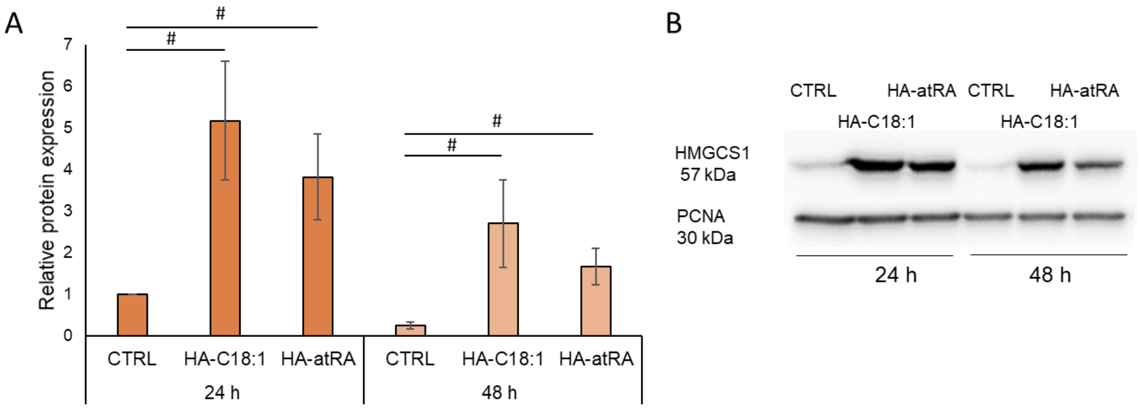

Figure S3. Protein expression of HMGCS1. HaCaT cells were treated with HA-atRA (100 µg/ml), HA-C18:1 (50 µg/ml) or left untreated. (a) Quantification of n=5 independent replicates. Bars represent the means±SD of signals related to those values of CTRL 24 h. # $p < 0.01$, t-test. (b) A representative Western blot of HMGCS1 and loading control PCNA.

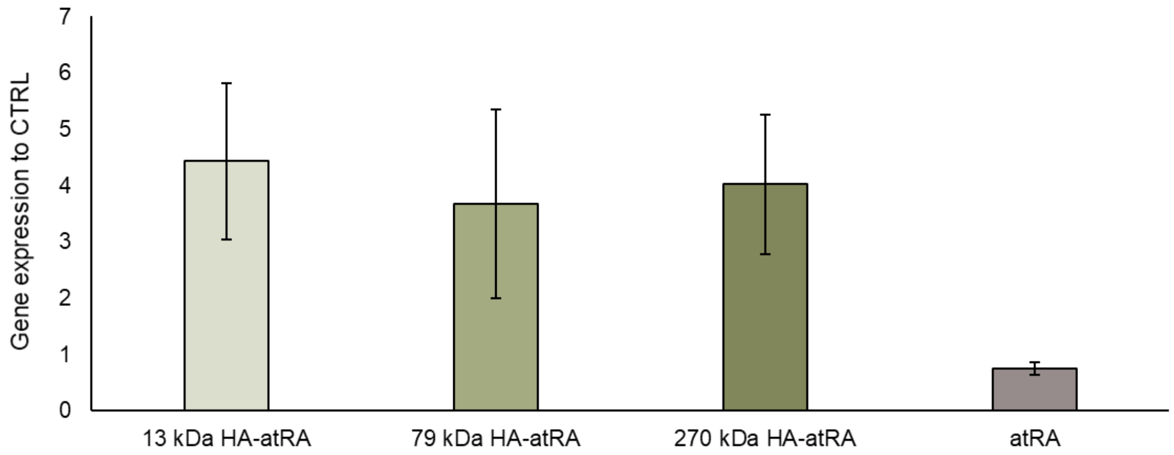

Figure S4. Gene expression of HMGCS1 is induced by different molecular sizes of HA-atRA. HaCaT keratinocytes were treated with 100 µg/mL of the HA derivatives (DS 5.5-6.3%) or atRA (4.86 µg/mL) for 24 hours. Gene expression was analyzed using qPCR. Mean±SD, n = 4. *$p<0.05$, t-test to untreated control.

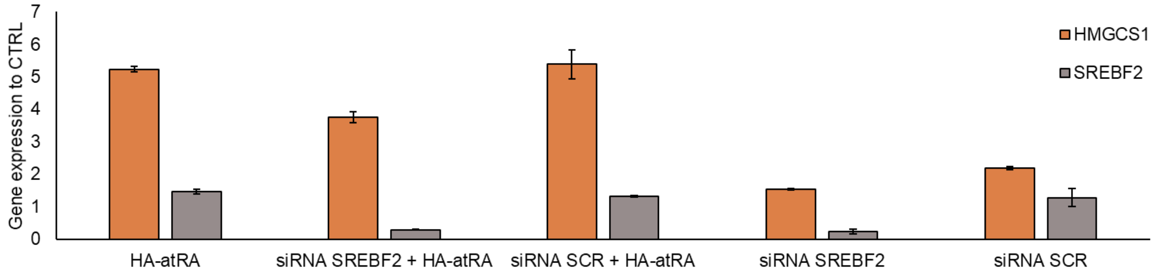

Figure S5. SREBF2 downregulation via siRNA. HaCaT keratinocytes were pretreated for 72 with siRNA against SREBF2, and subsequently, HA-atRA was added for 24 h. The gene expression of HMGCS1 and SREBF2 was measured using qPCR. The values were related to untreated control. Bars represent the means of n = 4 biological replicates. The error bars correspond to SD.
